# Control of Dichotomic Innate and Adaptive Immune Responses by Artery Tertiary Lymphoid Organs in Atherosclerosis

**DOI:** 10.3389/fphys.2012.00226

**Published:** 2012-07-06

**Authors:** Falk Weih, Rolf Gräbner, Desheng Hu, Michael Beer, Andreas J. R. Habenicht

**Affiliations:** ^1^Leibniz-Institute for Age Research, Fritz-Lipmann-InstituteJena, Germany; ^2^Institute for Vascular Medicine, Friedrich Schiller University, University Hospital JenaJena, Germany

**Keywords:** adaptive immune responses, artery tertiary lymphoid organs, atherosclerosis, autoimmunity, inflammation, stable plaque, vulnerable plaque

## Abstract

Tertiary lymphoid organs (TLOs) emerge in tissues in response to non-resolving inflammation such as chronic infection, graft rejection, and autoimmune disease. We identified artery TLOs (ATLOs) in the adventitia adjacent to atherosclerotic plaques of aged hyperlipidemic *ApoE*^−/−^ mice. ATLOs are structured into T cell areas harboring conventional dendritic cells and monocyte-derived DCs; B cell follicles containing follicular dendritic cells within activated germinal centers; and peripheral niches of plasma cells. ATLOs also show extensive neoangiogenesis, aberrant lymphangiogenesis, and high endothelial venule (HEV) neogenesis. Newly formed conduit networks connect the external lamina of the artery with HEVs in T cell areas. ATLOs recruit and generate lymphocyte subsets with opposing activities including activated CD4^+^ and CD8^+^ effector T cells, natural and induced CD4^+^ T regulatory (nTregs; iTregs) cells as well as B-1 and B-2 cells at different stages of differentiation. These data indicate that ATLOs organize dichotomic innate and adaptive immune responses in atherosclerosis. In this review we discuss the novel concept that dichotomic immune responses toward atherosclerosis-specific antigens are carried out by ATLOs in the adventitia of the arterial wall and that malfunction of the tolerogenic arm of ATLO immunity triggers transition from silent autoimmune reactivity to clinically overt disease.

## TLOs Arise in Chronic Non-Resolving Inflammation of Peripheral Tissues

**Figure 1:**
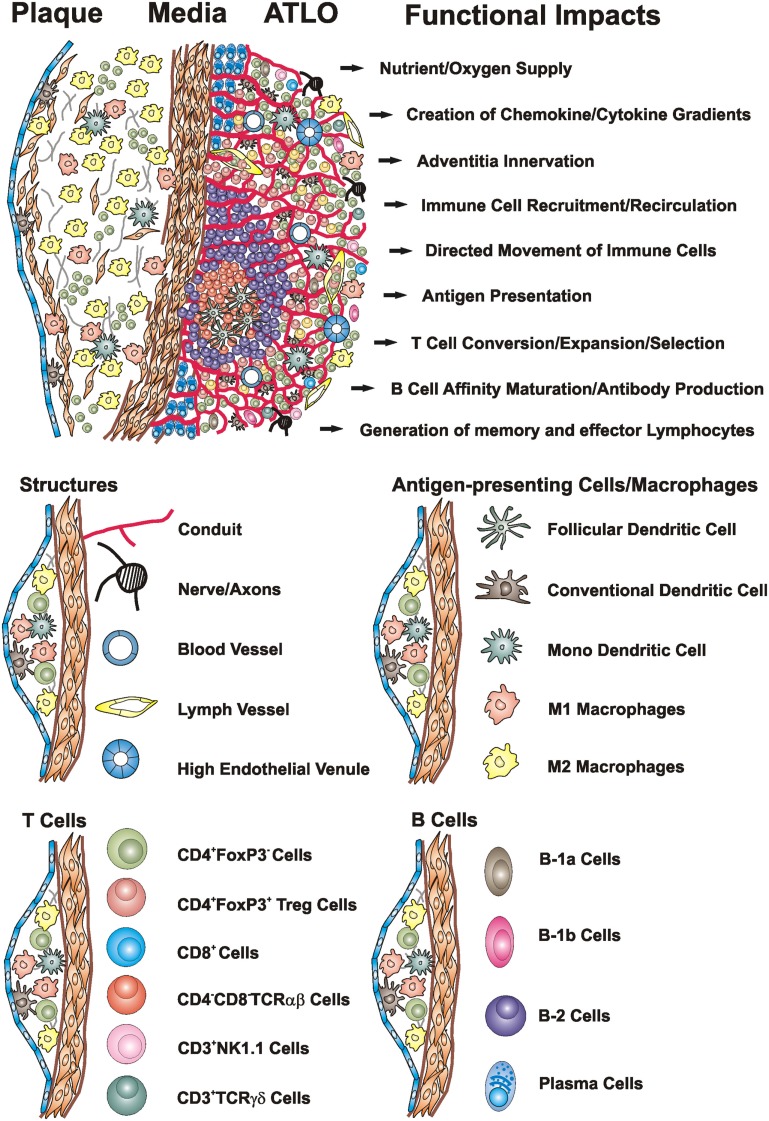
**Artery TLOs arise in the aorta adventitia of aged *ApoE−/−* mice adjacent to atherosclerotic plaques**. Cellularity, structures, and territoriality within the diseased arterial wall indicate that ATLOs organize inflammation-driven innate and adaptive immune responses in atherosclerosis: TLO conduits allow the creation of chemokine and cytokine gradients and the directed movement of T cells and DCs in lymph nodes (LNs) and spleen (Cyster, 2003; Nolte et al., 2003; Sixt et al., 2005; Bajenoff et al., 2006) and – by analogy – may maintain such gradients in the adventitia of diseased artery segments in ATLOs. ATLO conduits connect the external lamina of the arterial wall with HEVs in T cell areas and transport small MW molecules. In unpublished analyses, we observed a dense network of nerve axons within ATLOs but their impact remains unclear. Extensive newly formed blood vessels provide nutrient and oxygen supply. ATLOs strongly support T cell recruitment and recirculation whereas aberrant lymphangiogenesis may promote recruitments of DCs and other immune cells into the inflamed adventitia. ATLOs harbor several APCs including FDCs, cDCs, mDCs, and B cells. Several B-2 cells at different stages of differentiation and plasma cells are present in activated B cell follicles and the ATLO periphery, respectively. We also identified increased populations of innate B-1a and B-1b cells by FACS. Modified from Gräbner et al. (2009).

**Figure 2:**
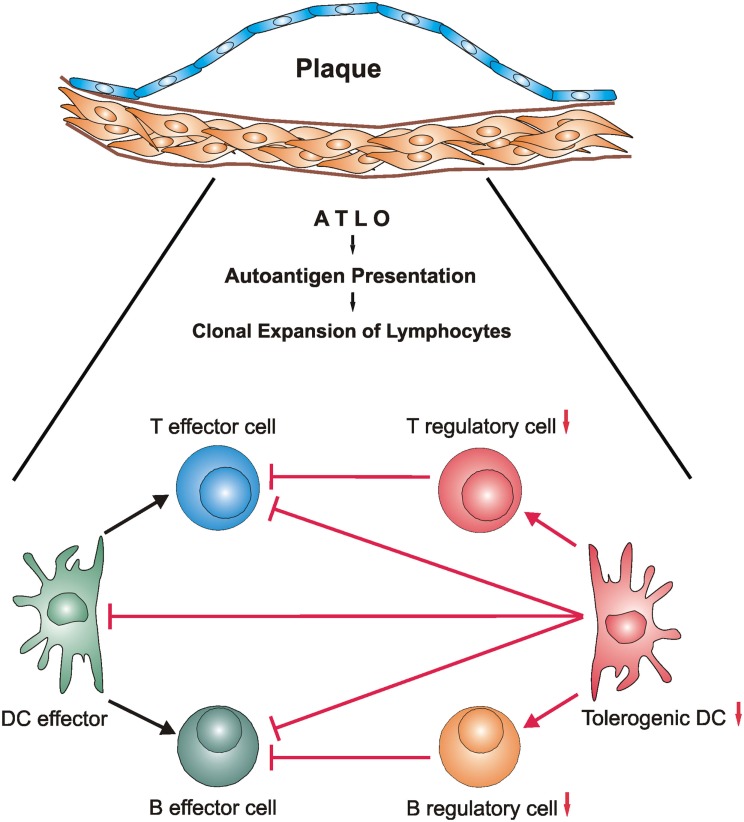
**The balance of ATLO antigen-specific immune cell effectors and their suppressor counterparts may be disturbed during development of unstable atherosclerotic plaques**. During long-lasting transmural arterial wall inflammation autoantigens may be generated within the diseased arterial wall and be presented by ATLO DCs (Steinman, 2012) and FDCs (Good-Jacobson and Shlomchik, 2010; Reizis et al., 2011a,b). This triggers generation of antigen-specific T and B cell effectors and their regulatory counterparts (Fontenot and Rudensky, 2005; Gräbner et al., 2009; Lund and Randall, 2010; Hu et al., unpublished) resulting in atherosclerosis-specific – yet clinically silent – autoimmune reactivity under steady state conditions. However, as ATLOs may organize the generation of pools of memory T and B lymphocyte subsets, a subsequent disturbance of the balance between autoreactive effectors and suppressors may hyperactivate the pro-atherogenic lymphocyte subsets and concomitantly compromise activity of their suppressors. Although mechanisms of unstable plaque formation are poorly understood in atherosclerosis, mechanisms of autoimmune injury of brain and other organs are better understood. By analogy, autoimmune T cells may contribute to the formation of vulnerable/unstable plaques during clinically significant atherosclerosis. Definition of molecular mechanisms of the emergence of autoreactivity from inflammation and the conversion of autoreactivity to autoimmune disease in atherosclerosis and other *bona fide* autoimmune diseases has a major potential to identify therapeutic targets. Note that innate immune cells and their dichotomic subtypes including innate B1-a/B1-b cells, natural T regulatory cells, and macrophage subtypes are not depicted in this scenario but aspects of their functional impact are subject to reviews in this series. Additional cell/cell interactions and marker expression of effectors or suppressors are not shown for ease of reading.

The immune system aims at identification and destruction of foreign antigens while preserving self (Shlomchik et al., 2001; Cyster, 2003; Drayton et al., 2003, 2006; Gommerman and Browning, 2003; Cupedo et al., 2004; Browning et al., 2005; Aloisi and Pujol-Borrell, 2006; Browning, 2006; Goodnow, 2007; Carragher et al., 2008; Shlomchik, 2008, 2009). To achieve this task, both innate and adaptive immune responses are intrinsically dichotomic in nature: elimination of antigen is associated with tissue inflammation with the potential to cause collateral damage leading to organ dysfunction. However, to protect the host from inflammation-triggered injury, the immune system employs a series of powerful strategies. It puts potent cells to work which implement versatile tools to constantly equilibrate the balance between destruction and protection. As long as this balance is efficiently maintained, organ damage can be avoided, and resolution of inflammation will be successful (Nathan and Ding, 2010). Generation of inflammatory versus anti-inflammatory leukocytes is achieved by cytokine-driven differentiation pathways creating innate and adaptive immune cell subsets in bone marrow, spleen, and peripheral tissues where antigens may arise. Mouse models have contributed much to our current understanding of human chronic inflammatory diseases but translation of experimental data to human diseases remains a major challenge (Van De Pavert et al., 2009; Blumberg et al., 2012; Roep et al., 2012). Innate immune cells are generated in highly localized tissue microdomains as shown for mouse Ly6C^+^ and Ly6C^−^ monocytes, M1- and M2-like tissue macrophages, immune response-promoting, and tolerogenic dendritic cells (DCs) as well as B-1a and B-1b cells (Libby, 2002; Alugupalli et al., 2004; Swirski et al., 2007; Tacke et al., 2007; Koltsova and Ley, 2011; Manthey and Zernecke, 2011). Likewise, the adaptive immune system generates antigen-specific T and B effector cells and their equally powerful tolerogenic antigen-specific T and B regulatory cell (Treg; Breg) counterparts (see below). Innate immune cells eliminate the antigen within minutes to hours before T and B cell activation, memory cell generation, clonal expansion, and affinity maturation are initiated. However, if antigen generation continues beyond the critical time window of 12–24 h and antigen eradication through innate immune cells fails, vigorous T and B cell responses are triggered (Mempel et al., 2004). Even under these conditions, an equilibrium between effector and tolerogenic lymphocyte responses prevents outbreak of clinical disease as shown by the presence of autoreactive T and B cells in a large percentage of the healthy population (Lang et al., 2005; Lopez-Diego and Weiner, 2008). However, chronic disturbance of this balance leads to tissue destruction and autoimmune injury (Nathan and Ding, 2010; Kuchroo et al., 2012). How tissue inflammation prompts the adaptive immune system to organize T and B cell immune responses through danger signal-activated antigen-presenting cells (APCs) is of major interest to understand principles of adaptive immunity, autoimmunity, and atherosclerosis (Mach et al., 1998; Ludewig et al., 2000; McLachlan and Rapoport, 2004; Goodnow, 2007; Herlands et al., 2008;Shlomchik, 2008, 2009; Galkina and Ley, 2009; Packard et al., 2009; Cheong et al., 2010; McInnes and Schett, 2011; Blumberg et al., 2012; Kuchroo et al., 2012; Roep et al., 2012; Sakaguchi et al., 2012; Steinman et al., 2012; Wekerle et al., 2012). In organ-specific autoimmune responses, T and B lymphocytes – together with activated stromal lymphoid tissue organizer cells – organize themselves as Tertiary lymphoid organs (TLOs) adjacent to or even within the inflamed target tissue (Moyron-Quiroz et al., 2004). Under these conditions, localized chronic inflammation prompts TLO neogenesis and *vice versa* TLOs amplify tissue inflammation. TLOs can therefore be viewed as hallmarks of organ-specific autoimmunity and – possibly – atherosclerosis (Figure 1). The immune system utilizes diverse strategies to detect and pick up particulate or soluble antigens. DCs constantly patrol peripheral tissues as sentinels to track down antigen-derived danger signals to carry antigen to secondary lymphoid organs (SLOs) to initiate T cell responses (Steinman, 2012) while follicular dendritic cell (FDCs) bind soluble antigens as immune complexes to initiate and organize B cell affinity maturation. What, then, may be the advantage of TLO neogenesis within the target organ of autoimmunity versus antigen presentation in SLOs? First, in TLOs, autoantigen can be presented within close distance of its generation avoiding dilution thereby lowering the antigen threshold to trigger an adaptive T cell response. Second, the cytokine environment of TLOs may also stimulate recruitment of blood monocytes and generate fully effective monocyte-derived DCs (mDCs) in addition to conventional dendritic cells (cDCs; Lee et al., 2007; Randolph et al., 2008a,b; Cheong et al., 2010; Choi et al., 2011). Third, immune complexes with unprocessed antigen can gain access to TLOs where they bind to FDCs within germinal centers (GCs) at higher concentrations compared to FDCs in the more distant SLOs (Kratz et al., 1996; Mackay and Browning, 1998; Stott et al., 1998; Kim et al., 1999; Luther et al., 2000; Weyand et al., 2001; Itano and Jenkins, 2003; Kosco-Vilbois, 2003; Moyron-Quiroz et al., 2004; Allen et al., 2007; Lee et al., 2007; Timmer et al., 2007; Lund and Randall, 2010; Sweet et al., 2011). Permissive conditions for SLO and TLO formation arise in the connective tissues when *lymphoid tissue organizer* cells interact with immune cells termed *lymphoid tissue inducer* cells (Roozendaal and Mebius, 2011). This occurs during embryogenesis at predetermined sites to generate lymph nodes and gut-associated lymphoid tissues or at diverse locations in adult organisms to initiate the formation of TLOs (Cupedo et al., 2004). Thus, unlike SLOs, TLOs function as effective and, depending on the conditions, transient organizers of adaptive immune responses in chronically inflamed tissues. Why has it not be possible to clearly define functional impacts of TLOs in any autoimmune disease? There is ample evidence that breakdown of tolerance is required to convert clinically silent *autoimmune reactivity* to *autoimmune disease* and this breakdown of tolerance may not occur during TLO formation *per se* (see below Figure 2). Importantly, breakdown of tolerance primarily occurs in the periphery in tissue microdomains (Shlomchik et al., 2001; Shlomchik, 2008, 2009; Good-Jacobson and Shlomchik, 2010).

Under conditions of tolerance breakdown, identification of the triggers of lymphocyte activation, their emigration from SLOs or TLOs, and mechanisms of lymphocyte homing to attack the antigen-specific targets in human disease remain important issues of autoimmune disease research including atherosclerosis (Hansson et al., 1989; Lang et al., 2005; Galkina and Ley, 2009; Hermansson et al., 2010; Hansson and Lundberg, 2011). Indeed, TLOs are closely associated with various autoimmune diseases but their presence does not seem to be sufficient to trigger organ injury. To understand autoimmune-triggered organ dysfunction, it is crucial to strictly distinguish autoimmune reactivity from autoimmune disease. A major function of TLOs is to organize B cell immunity: B cells reside, act, proliferate, and undergo affinity maturation locally using the inflammatory survival niches and lymphorganogenic chemokines such as CXCL13, CCL21, and lymphotoxin (Schroder et al., 1996; Luster, 1998; Stott et al., 1998; Kim et al., 1999; Gräbner et al., 2009; Sweet et al., 2011). Thus, the initiation of autoimmune disease is the result of a multistep process in which TLO neogenesis appears to be required but is not sufficient: Additional events including toll-like receptor activation and breakdown of tissue barriers such as the blood brain barrier in multiple sclerosis (see below) are needed to trigger overt autoimmune disease (Cole et al., 2011; Hansson and Lundberg, 2011). To facilitate local adaptive immune responses, TLOs generate and assemble conduits, HEVs, and lymph vessels to boost T and B cell recruitment and to promote their movement within T cell areas or B cell follicles as shown for ATLOs. These structures enhance the probability for TCR- or BCR-carrying lymphocytes to find their cognate antigen close to its generation. In ATLOs, we observed that HEV neogenesis and maintenance is dependent on ongoing lymphotoxin β receptor (LTβR) signaling (Figure 1). However, TLOs differ markedly from SLOs in structure, cellularity, and function in several important aspects. Although there is evidence from human autoimmune diseases indicating that TLOs mount specific T and B cell immune responses toward self-antigens (Fütterer et al., 1998; Kim et al., 1999; Ettinger et al., 2001; Gommerman and Browning, 2003; Cupedo et al., 2004; Browning, 2006; Lee et al., 2006), major issues of their formation and functional impact on disease progression remain to be explored: (*i*) How do innate immune cells, i.e., activated monocytes/macrophages, neutrophils, mast cells, DCs, and lymphoid tissue inducer cells prompt adaptive immune responses within the target tissue (Tellides et al., 2000; Lopez-Diego and Weiner, 2008)? (*ii*) Does the formation of TLOs require the presence of an antigen/autoantigen or is autoimmunity a late event after TLOs have formed (Kosco-Vilbois, 2003; Thaunat et al., 2005; Allen et al., 2007)? (*iii*) How does overt autoimmune disease arise from clinically quiescent autoimmune reactivity (Lang et al., 2005? (*iv*) Are immune responses in TLOs and SLOs distinct (Farez et al., 2009)? (*v*) What is the relative share of autoreactive T and B cells versus innate immune cells to direct tissue destruction? (*vi*) How is the apparent equilibrium between effector and tolerogenic arms of the immune system in TLOs maintained under steady state conditions and how is it disturbed during disease progression and/or disease relapse (Kosco-Vilbois, 2003; Hansson, 2005;Ait-Oufella et al., 2006, 2010; Herlands et al., 2008)? Answers to these questions will be crucial to develop immune-based therapies to treat autoimmune disorders and atherosclerosis. In this review, we focus on atherosclerosis- and hyperlipidemia-dependent ATLO neogenesis in aged *ApoE*^−/−^ mice (Figure 1).

## The Demanding Task to Distinguish Chronic Inflammation, Adaptive Immune Responses, Autoimmune Reactivity, and Autoimmune Disease in Atherosclerosis

Only few human autoimmune diseases, i.e., Hashimoto’s thyroiditis, Grave’s disease, and myasthenia gravis, meet the *direct Witebsky postulates* of autoimmune diseases (Witebsky et al., 1957): Isolation of a *pathogenic autoantigen*, isolation of a B cell clone with *exquisite specificity for autoantigen*, demonstration that B cell clones produce *pathogenic autoantibodies* in experimental transfer experiments, *T cells responding to pathogenic autoantigen* in an autologous mixed lymphocyte reaction, isolation of *T cells carrying a TCR with specificity for autoantigens*, and cloning of *pathogenic T cells able to transfer autoimmune disease to another individual* (Olsson et al., 1990; Rose and Bona, 1993; McLachlan and Rapoport, 2004; Rose, 2006). In these classical organ-specific autoimmune diseases, pathogenic autoantibodies against thyroglobulin, thyroperoxidase, or nicotinic acetylcholine receptors have been demonstrated to trigger the disease (Ettinger et al., 2001; Carragher et al., 2008). For most clinically important human autoimmune diseases, i.e., rheumatoid arthritis, diabetes mellitus type I, and multiple sclerosis, however, Witebsky postulates are not completely met and evidence for autoimmune-triggered organ damage is strong yet circumstantial. Moreover, much of the evidence that chronic inflammatory human diseases are associated with a significant autoimmune component comes from animal models (Olsson et al., 1990; Ettinger et al., 2001; McLachlan and Rapoport, 2004; Lee et al., 2006; Zhou et al., 2006; Timmer et al., 2007; Coppieters et al., 2012). Interestingly, the *American Autoimmune Related Diseases Association* does not list atherosclerosis as an autoimmune or an autoimmune related disease (http://www.aarda.org). The view that atherosclerosis is associated with autoimmune responses, a view that we favor (Grundtmann et al., 2011), is largely derived from circumstantial evidence such as the presence of autoantibodies directed against presumptive autoantigens such as heat shock protein 60 or oxidized LDL (Van Puijvelde et al., 2007), and more recently by the demonstration that DC-like cells are present in atherosclerotic plaques (Wick et al., 1997, 2004; Lusis, 2000; Glass and Witztum, 2001; Libby, 2002; Witztum, 2002; Hansson, 2005; Tedgui and Mallat, 2006; Steinberg and Witztum, 2010; Choi et al., 2011; Libby et al., 2011; Weber and Noels, 2011). However, autoreactive T and B cells and autoantibodies can be detected in a large percentage of the healthy population and their presence often does not correlate with clinically significant autoimmune diseases. Therefore, when compared to well characterized human autoimmune disorders, our understanding of autoimmunity in atherosclerosis is at an early stage. Furthermore, unlike multiple sclerosis (Lopez-Diego and Weiner, 2008), diabetes mellitus type I (Lee et al., 2006), and rheumatoid arthritis (Aloisi and Pujol-Borrell, 2006), in which immune-based therapies have entered routine clinical practice, similar therapies for atherosclerosis are not in sight. Why is progress into the autoimmune origin of atherosclerosis lagging behind? One reason may be that it has been an extremely challenging task to isolate pathogenic autoantibodies for most human diseases including atherosclerosis because chronic inflammation is associated with the generation of multiple autoantigens, a phenomenon referred to as *epitope spreading* (Miller et al., 1997; McMahon et al., 2005). This makes identification of *disease-causing* as opposed to irrelevant *bystander* autoantigens a complex task. It has also been difficult to understand the mechanisms underlying the clinical courses of chronic inflammatory diseases such as relapses, long periods of intermittent latency periods, and periods of violent progressive phases. Unfortunately, mouse models rarely mimic these events. However, the existence of polyclonal T cell and antibody responses that may initiate a vicious circle of immune injury and inflammation is supported by a large body of circumstantial evidence in many of these diseases including atherosclerosis (Olsson et al., 1990; Ettinger et al., 2001; McLachlan and Rapoport, 2004; Browning, 2006; Lee et al., 2006; Herlands et al., 2008; Gräbner et al., 2009; Good-Jacobson and Shlomchik, 2010; Coppieters et al., 2012; Kuchroo et al., 2012; Roep et al., 2012). Dramatic antigen spreading during the primary progressive and relapsing-remitting phases of multiple sclerosis exemplifies the daunting challenges to identify culprit autoantigens in atherosclerosis (Quintana et al., 2008, 2012). Other reasons for the slow progress into autoimmunity of atherosclerosis are its multistep pathogenesis, the strong association with age (the major but least understood risk factor for atherosclerosis, see below), its complex risk factor profile, the crucial influence of environmental factors on its progression, and the lack of a robust mouse model. TLOs and ATLOs share functional and structural features with SLOs including separate T cell areas and B cell follicles yet important differences are apparent. Although the wild-type mouse adventitia contains a network of lymph vessels, the newly formed ATLO lymph vessels show aberrant features. Their distended lumen is congested with a large number of leukocytes whose movement and/or impaired transendothelial migration is reminiscent of tumor lymphangiogenesis (Oliver, 2004; Furtado et al., 2007; Gräbner et al., 2009). It will be of interest to determine the mechanisms and functional implications of these abnormalities and analyze which type of immune cells migrate through the aberrant ATLO lymph vessels. Recently, we analyzed the phenotypes of immune cells by immunofluorescence and flow cytometry. ATLOs contain large numbers of plasma cells which are rare in LNs or spleen. The origin of these plasma cells in diseased arteries is not known but in analogy to rheumatoid arthritis they may derive from activated B cell follicles (Kim et al., 1999). A major question regarding the organization of the B cell adaptive immune response is the role of FDCs in the B cell follicles. It has been shown that FDCs and affinity maturation of B cells require ongoing LTβR signaling as well as the presence of antigen (Schroder et al., 1996; Fütterer et al., 1998; Mackay and Browning, 1998; Endres et al., 1999; Gommerman and Browning, 2003; Kosco-Vilbois, 2003; Victoratos et al., 2006). These data raise the important possibility that ATLO FDCs present arterial wall-derived autoantigens and that B-2 cells undergo affinity maturation giving rise to memory and/or plasma cells. Moreover, we recently observed that ATLOs contain significant numbers of B-1 cells that predominantly belong to the B-1b cell subtype (Srikakulapu et al., unpublished observations). These data indicate that ATLOs not only promote T cell-dependent immune responses but also T cell-independent humoral immune responses that are carried out by natural antibody-producing B-1 cells within the diseased arterial wall. To understand the immune responses in ATLOs better we have used laser capture microdissection-based microarray analyses of ATLOs and compared transcriptomes of ATLOs directly with those of the draining renal LNs (Beer et al., 2011; Hu et al., unpublished data). When ATLOs were compared with the adventitia of wild-type aorta large numbers of immune response-regulating genes were acquired. These ATLO transcriptomes resembled those of SLOs, yet inflammation-regulating genes were expressed at significantly higher levels in ATLOs compared to LNs. Though characterization of macrophage subtypes in atherosclerosis has progressed during recent years. ATLO macrophage subtypes have not yet been characterized in detail.

## Search for a Mouse Model of Atherosclerosis Autoimmune Responses

As there are severe restrictions to study mechanisms of autoimmune diseases in humans, mouse models have been essential to understand their basis, complexity, and multistep features (Goodnow, 2007; Kuchroo et al., 2012; Roep et al., 2012). While each of the mouse models has its own limitations, there is a need to develop new models and experimental criteria in which distinct aspects of autoimmunity and the triggers of tolerance breakdown can be addressed (Wekerle et al., 2012). The well established mouse models *experimental autoimmune encephalomyelitis* (EAE) and *collagen-induced arthritis* (CIA) have guided the implementation of current therapeutic strategies in the clinic and some have even entered routine clinical practice (Lopez-Diego and Weiner, 2008; McInnes and Schett, 2011; Blumberg et al., 2012; Kuchroo et al., 2012). These examples show the enormous importance of suitable mouse models for autoimmune disease research. Although several attempts have been reported to address aspects of atherosclerosis autoimmunity in mice, it is our view that there is currently no robust mouse model to faithfully examine atherosclerosis autoimmune responses (Rosenfeld et al., 2000; Grundtmann et al., 2011; Libby et al., 2011). In this context, it is informative to briefly review the structure and cellularity of atherosclerotic plaques in humans in comparison to mouse models. Human atherosclerosis is clinically silent for long periods of time. It is now established that plaque growth is necessary but not sufficient to cause disease. An increase in plaque size is compensated by extensive outward media remodeling preventing tissue infarcts during early atherosclerosis. Observational studies of human coronary artery disease across all age groups show that asymptomatic fibroatheromatous plaque buildup can continue for decades without developing into clinically overt disease (Rekhter, 2002; Virmani et al., 2005). Initiation of disease requires development of vulnerable plaques as evidenced by fibrous cap thinning, enlargement of the necrotic core, macrophage activation, plaque neoangiogenesis, plaque rupture, bleeding, and thrombosis. However, the mechanisms how stable plaques undergo prototypical alterations to become vulnerable plaques are not well understood (Virmani et al., 2005) but autoimmune T cells have been held responsible (Hansson, 2005). In contrast to human disease, early inflammatory cell infiltrates of mouse atherosclerotic plaques consist of cDCs, mDCs, M1- and M2-type macrophages, CD4^+^ and CD8^+^ T cells, and proliferating smooth muscle cells covered by a stable fibrous cap. In the majority of reports, adolescent mice as young as 5–9 weeks are maintained on atherogenic Western-type diets for comparably short periods of time. Despite severe atherosclerosis, compensatory arterial wall remodeling ensures blood flow and even severely diseased mouse coronary arteries are never associated with tissue infarcts, even in aged *ApoE*^−/−^ mice (Gräbner et al. unpublished; see below). The disease readout is often the size of the plaque relative to the size of the media, i.e., the intima/media ratio and/or the area covered by macrophages/foam cells. While determination of intima/media ratios is useful to examine plaque growth it may not be very helpful to examine clinically significant disease. It is therefore possible that if autoimmune responses are to be identified in atherosclerosis, the value of current mouse models appears to be limited. However, some features of vulnerable plaques and myocardial infarcts have been induced in *ApoE*^−/−^ mice under distinct experimental conditions (Caligiuri et al., 1999). For instance, supplementing the Western diet with cholate induces vulnerable-like plaques but it also causes systemic inflammatory disease and organ damage of liver, skin, kidney, and myocardium. One important parameter seems to be age which has been shown to generate several but not all parameters of vulnerable plaques in the innominate artery of *ApoE*^−/−^ mice (Rosenfeld et al., 2000; Roncal et al., 2010). We observed that the infiltrate of early atherosclerotic plaques in the aorta is replaced by an increasing share of extracellular matrix and extensive outward media remodeling during aging of *ApoE*^−/−^ mice. Although the media is infiltrated by plaque leukocytes, vulnerable plaques – i.e., plaque rupture, thrombosis, and myocardial infarction – cannot be observed even in mice as old as 120 weeks. Thus, young mice though useful to delineate aspects of plaque growth and adaptive T cell immune responses (Cheong et al., 2010; Choi et al., 2011; Hansson and Hermansson, 2011) may be less suitable to examine clinical disease or autoimmune responses.

## ATLOs Emerge in the Adventitia in Response to Plaque Inflammation in Aged *ApoE*^−/−^ Mice and Indicate Robust Autoimmune T and B Cell Responses

As discussed above, atherosclerosis complies with indirect and circumstantial lines of evidence that support a role of autoimmunity in this disease. These include disease-suppressing effects of natural antibodies, oligoclonal T cell expansion toward potential autoantigens, protective roles of Tregs, pro-, and anti-atherogenic impacts of distinct B cell subtypes, and inhibition of plaque growth by vaccination (Mach et al., 1998; Ludewig et al., 2000; Zhou et al., 2000; Glass and Witztum, 2001; Caligiuri et al., 2002; Major et al., 2002; Witztum, 2002; Binder et al., 2003, 2005; Schiopu et al., 2004; Ait-Oufella et al., 2006; Tedgui and Mallat, 2006; Michel et al., 2007; Randolph et al., 2008a,b; Packard et al., 2009; Geissmann et al., 2010a,b; Hermansson et al., 2010; Steinberg and Witztum, 2010; Libby et al., 2011; Manthey and Zernecke, 2011; Weber and Noels, 2011). However, major issues of atherosclerosis immune responses and autoimmunity remain unresolved: (*i*) Where are adaptive and autoimmune responses organized? (*ii*) Which immune cells participate in arterial wall remodeling during different stages of the disease? (*iii*) Is the adaptive immune response in atherosclerosis systemic or organ-specific? (*iv*) Are there periods of heightened immune activation and what are the triggers of relapses and violent immune cell activities in acute coronary syndromes? (*v*) What are the contributions of innate immunity carried out by subtypes of blood-derived monocyte/macrophages and foam cells, of DC subtypes, and of B-1 cells? (*vi*) Are there antigen-specific CD4^+^ and/or CD8^+^ effector T cells directly targeting structures of the arterial wall? (*vii*) What causes dysfunction of the balance between effector cells and tolerogenic DCs, Tregs, and Bregs? And, most importantly (*viii*) what is the nature of the disease-triggering autoantigen(s)? It has been widely assumed that T cell responses are organized either in atherosclerotic plaques or in SLOs (Lusis, 2000; Libby, 2002; Witztum, 2002; Wick et al., 2004; Tedgui and Mallat, 2006; Grundtmann et al., 2011; Hansson and Hermansson, 2011). Until recently, the leukocyte infiltrates in the adventitia during atherogenesis have not been characterized in detail and adventitial inflammation has largely been regarded as an epiphenomenon with little relevance to disease progression although the role of adventitial *vasa vasora* has received some attention (Virmani et al., 2005). Importantly, the accumulation of leukocytes in the adventitia during atherogenesis has been noted decades ago. *Small round cell infiltrates* were reported in the adventitia of patients afflicted with coronary artery disease. The adventitia has been the subject of original reports and its potential role in atherogenesis has been reviewed (Gerlis, 1956; Schwartz and Mitchell, 1962; Stefanadis et al., 1995; Scott et al., 1996; Walton et al., 1997; Kwon et al., 1998; Labinaz et al., 1999; Pels et al., 1999; Herrmann et al., 2001; Houtkamp et al., 2001; Okamoto et al., 2001; Zhao et al., 2004; Moos et al., 2005; Cheema et al., 2006; Galkina et al., 2006; Michel et al., 2007; Watanabe et al., 2007). Local humoral immune responses have been suggested to be organized by atherosclerosis-associated adventitial lymphoid aggregates containing B cells and FDCs (Houtkamp et al., 2001). The detailed characterization of ATLO T and B cells and of the ATLO microarchitecture provides strong evidence for an autoimmune contribution during late stage atherosclerosis: ATLOs show striking similarities to TLOs in prototypic organ-specific autoimmune disorders including immune cell subtypes, distinct structures such as HEVs, and a high degree of territoriality adjacent to the target organ (for atherosclerosis adjacent to atherosclerotic plaques; Moos et al., 2005; Galkina et al., 2006; Galkina and Ley, 2009; Gräbner et al., 2009). Our studies in *ApoE*^−/−^ mice during aging revealed that adventitial T cell infiltrates occur in parallel with the formation of intima plaques in the innominate artery and in the abdominal aorta, generating large immune cell aggregates after the age of 52 weeks. ATLOs are now among the best characterized TLOs in any chronic inflammatory disease. Remarkably, the number of T cells in the adventitia of diseased aorta segments exceeds the number of plaque T cells by a factor of >80-fold (Moos et al., 2005). Subsequently, B cell aggregates form and DCs as well as plasma cells localize in the ATLO periphery (Zhao et al., 2004; Moos et al., 2005; Gräbner et al., 2009). Thus, while early atherosclerosis is associated with significant T cell infiltrates in the intima, T cell density in plaques decreases over time whereas it dramatically increases in the adventitia during aging. Furthermore, B cells, which are absent in the normal aorta and in atherosclerotic plaques, form aggregates during intermediate stages of ATLO neogenesis, whereas advanced stages of ATLOs are characterized by large B cell follicles and ectopic GCs containing FDC networks with proliferating B cells (Figure 1). Importantly, FDCs indicate adaptive B cell responses, antigen-specific B memory cell formation, and affinity maturation of B cells. The presence of FDCs in ATLOs and of proliferating B cells in ATLO GCs provide strong evidence for a robust antigen-specific autoimmune response within the diseased arterial wall adventitia (Figure 2). Delineation of ATLO cellularity suggests that the diseased artery is capable of organizing both T cell and B cell autoimmune responses and that these responses are not observed in young animals. In *ApoE*^−/−^ mice, we observed preferential formation of ATLOs in the upper portion of the abdominal aorta but occasionally also in coronary and pulmonary arteries, in the adventitia of the brachiocephalic trunk, in the adventitia of the innominate artery, in aortic valves, and rarely in the myocardium. Apparently, the connective tissue of the adventitia of the abdominal aorta provides particularly permissive conditions for ATLO formation but the molecular basis for this preferred location is not known. We never observed ATLO neogenesis in artery segments that are not burdened by advanced atherosclerotic plaques in the intima nor did we observe ATLOs in the intima. However, the formation of large and advanced plaques is not sufficient to trigger ATLO formation as atherosclerosis in *ApoE*^−/−^ mice begins and is most advanced in the aortic arch where ATLOs can only rarely be observed. The occurrence of ATLOs in the abdominal aorta adventitia is reminiscent of TLO formation in the meninges in multiple sclerosis. TLO formation requires long-lasting interactions between immune cells and lymphoid tissue organizer cells in multiple feedback loops involving a series of hematopoietic and connective-tissue-derived cytokines (Zinkernagel et al., 1997; Mackay and Browning, 1998; Luther et al., 2000; Weyand et al., 2001; Mebius, 2003; Cupedo and Mebius, 2005; Ware, 2005; Timmer et al., 2007; Van De Pavert et al., 2009; Roozendaal and Mebius, 2011). In atherosclerosis, lymphoid tissue organizer cells may arise from media smooth muscle cells as indicated by *in vitro* studies. Interestingly, smooth muscle cells, but not endothelial cells, stimulated *in vitro* with agonistic anti-LTβR antibodies acquire features of lymphoid tissue organizer cells including induction of the lymphorganogenic chemokine CXCL13 (Lötzer et al., 2010). Alternatively, myofibroblasts in the adventitia of large arteries during atherogenesis may act as lymphoid tissue organizer cells to support ATLO formation. A more detailed knowledge about development and function of ATLOs will likely help to better understand both innate and adaptive atherosclerosis immunity.

## The Territoriality of Atherosclerosis Immune Responses: Plaque and/or Adventitia, Organ-Specific, and/or Systemic?

Since the ‘response to injury hypothesis’ proposed four decades ago emphasized the role of smooth muscle cells for atherosclerosis progression (Ross and Glomset, 1973, 1976a,b), it is now established that adaptive immune responses contribute to disease progression. The majority of investigators assume that these responses are carried out in the intima of the diseased arterial wall and/or in SLOs (Wick et al., 1997, 2004; Lusis, 2000; Zhou et al., 2000; Libby, 2002; Witztum, 2002; Tedgui and Mallat, 2006; Hansson and Hermansson, 2011; Weber and Noels, 2011). Immune response-regulating cells in atherosclerosis in plaques have also been termed *vascular-associated lymphoid tissue* (Wick et al., 1997, 2004; Grundtmann et al., 2011) and it has been proposed that epitopes of heat shock protein Hsp60 or oxLDL or LDL are the culprit autoantigens (Paulsson et al., 2000; Schiopu et al., 2004; Randolph et al., 2008a,b; Packard et al., 2009; Hermansson et al., 2010; Klingenberg et al., 2010; Steinberg and Witztum, 2010; Manthey and Zernecke, 2011). Several immune cells are systemically altered by hyperlipidemia in mouse models (Swirski et al., 2007; Tacke et al., 2007). Under conditions of acute myocardial infarction, spleen monocytes are rapidly mobilized into the heart (Leuschner et al., 2012). However, it is much less clear whether there are systemic alterations in the number, activation, or lymphocyte subset composition during atherogenesis in hyperlipidemic mice maintained under normal mouse chow. Thus, despite extensive efforts it is still largely unclear whether atherosclerosis is associated with organ-specific or systemic adaptive and/or autoimmune reactions. Systemic vaccination approaches using various presumptive autoantigens including LDL, oxLDL, and heat shock protein in hyperlipidemic mice resulted in attenuation or acceleration of disease severity providing circumstantial evidence for systemic immune activity. Moreover, T cell-oriented intervention in mice vaccinated with oxLDL also led to a decrease in atherosclerosis severity but the mice generated a marked T cell response against LDL rather than oxLDL epitopes (Hermansson et al., 2010; Klingenberg et al., 2010). Depletion of T regulatory cells using anti-CD25 antibodies in hyperlipidemic mice increased atherosclerosis pathology and anti-CD20 antibody-induced B cell depletion also resulted in a decrease in disease severity implicating anti-atherogenic actions of natural T regulatory (nTregs) and pro-atherogenic actions of B cell subsets (Ait-Oufella et al., 2006, 2010). All these observations provide circumstantial evidence that T and B cell responses affect atherosclerosis systemically and dichotomically. A number of recent studies including our own are more consistent with the view, however, that atherosclerosis is an organ-specific disease. This suggestion is based on the cellular composition and structure of ATLOs in the adventitia of *ApoE*^−/−^ mice (Figures 1 and 2). In addition, age has been associated with some autoimmune diseases (Weyand et al., 2001; Goronzy and Weyand, 2003; Linton and Dorshkind, 2004; Wick et al., 2004; Lindström and Robinson, 2010) and age is the most important though least understood risk factor for atherosclerosis. As we observed a large increase in adventitial immune cell accumulation in aged *ApoE*^−/−^ mice, the possibility that immune senescence contributes to atherosclerosis deserves attention (Linton and Dorshkind, 2004). The first initial stages of ATLOs were noted at around 52 weeks of age with preferential formation in the upper abdominal aorta where aortic aneurysms are formed (Zhao et al., 2004). Fully developed ATLOs including activated GCs and FDCs emerged between 52 and 78 weeks of age in aorta segments burdened with atherosclerotic plaques. Atherosclerosis becomes clinically significant only when the late stages are reached, often after short periods of violent progression of plaque growth, leading eventually to plaque instability and rupture (see above). Unfortunately, such stages are difficult to study in mouse models as hyperlipidemic mice have a normal lifespan and never develop myocardial infarction under steady state conditions and normal mouse chow, indicating that poorly understood secondary events are required to initiate clinically severe disease (Figure 2). For example, it is not known whether immune senescence is involved and to which extent aging of stromal cells such as media smooth muscle cells participates in atherosclerosis progression (Lindström and Robinson, 2010; Roozendaal and Mebius, 2011).

## Autoimmune Reactivity is not Sufficient to Initiate Autoimmune Disease

Several caveats merit consideration regarding a pathogenic role of ATLOs in atherosclerosis. First, TLOs as organizers of adaptive immune responses generate both effector lymphocytes and concomitantly their immunosuppressive counterparts in an apparent equilibrium (Figure 2). It is therefore not clear whether, when, and how the balance between effector and tolerogenic lymphocytes is disturbed to cause autoimmune tissue injury. Second, the relative contribution of inflammation and specific lymphocyte responses to tissue injury have not yet been defined. It is possible that silent disease phases are characterized by a well-tuned balance between effector and suppressor activities and that disease relapses and rapid progression are caused by activation of effector lymphocytes and/or inhibition of suppressor activities. The important question what triggers this lymphocyte activation still has to be answered but molecular mimicry, i.e., crossreactivity between bacterial or viral epitopes with epitopes of self-antigens, merits attention (Olson et al., 2001; Binder et al., 2003). A large body of evidence supports the assumption that atherosclerosis involves T and B cell immune responses that promote or inhibit plaque growth (Ramshaw and Parums, 1990;Wick et al., 1997, 2004; Mach et al., 1998; Paulsson et al., 2000; Zhou et al., 2000; Weyand et al., 2001; Major et al., 2002; Schiopu et al., 2004; Zhao et al., 2004; Moos et al., 2005;Ait-Oufella et al., 2006, 2010; Galkina et al., 2006; Niessner et al., 2006; Van Puijvelde et al., 2007; Hermansson et al., 2010; Kyaw et al., 2010; Steinberg and Witztum, 2010; Grundtmann et al., 2011). The dichotomic nature of organ-specific autoimmune diseases is well established for EAE as shown by the various immune cell subsets in meningeal TLOs (Aloisi and Pujol-Borrell, 2006). The presence of discrete T cell areas and FDC networks within activated GCs of ATLOs provides the first indirect evidence that the transmural inflammation of the arterial wall generates atherosclerosis-specific antigen(s) that may trigger specific T cell and B cell immune responses. Similar to SLOs, organization of the ATLO immune response is dependent on the LTβR (Alimzhanov et al., 1997; Endres et al., 1999; Kosco-Vilbois, 2003; Weih and Caamano, 2003; Oliver, 2004; Ware, 2005; Victoratos et al., 2006; Allen et al., 2007; Furtado et al., 2007; Lee et al., 2007; Gräbner et al., 2009). Importantly, lymphotoxin signaling through the LTβR is not only essential for the maintenance of FDC networks and for immunoglobulin affinity maturation (Fütterer et al., 1998; Gommerman and Browning, 2003), but it also regulates autoimmune disease in a mouse model of diabetes mellitus type I (Ettinger et al., 2001; Lee et al., 2006). We speculate that ATLOs contain pro-atherogenic and anti-atherogenic T cells as well as B cells in apparent equilibria, raising the important question how this balance might be disturbed during disease progression (Figures 1 and 2). The complexity of the immune response in atherosclerosis precludes for now to predict what these mechanisms may be. For instance, the tolerogenic arms of the atherosclerosis adaptive immune response might be compromised. To examine this possibility at a cellular level, further studies of arterial wall Treg cells and of B-1 cells should be performed in addition to cDCs which have recently been shown to exert a protective role during early stages of atherosclerosis in young *ApoE*^−/−^ mice (Choi et al., 2011). Marked increase of local lymphocyte recirculation, recruitment, functional conduit formation, blood vessel, and HEV neogenesis as well as pathological lymphangiogenesis are all indicative for autoimmune reactivity but not consequentially of autoimmune disease or arterial wall injury. It is recognized that autoimmune diseases in humans develop in separable steps and that only in the late stages, when tolerance against autoantigen(s) is breaking down, conversion to explicit self-reactivity associated with debilitating tissue destruction can be observed (Lang et al., 2005). In the respective EAE and CIA mouse models for human multiple sclerosis (TLOs form in the meninges) and rheumatoid arthritis (TLOs form in the synovial membrane and bone adjacent to the joint cartilage), the disease appears to go through phases of relapses and attenuation and relapses are often triggered by infections and it has been suggested that toll-like receptor signaling may be involved in disease progression (Leadbetter et al., 2002; Lang et al., 2005; Herlands et al., 2008; Farez et al., 2009; Garin et al., 2010).

## Concluding Remarks

The autoimmune hypothesis of atherosclerosis is based on indirect and circumstantial evidence. Much work is needed to obtain more direct evidence for participation of autoreactive lymphocytes in the clinically important stages of the disease when stable clinically silent atherosclerosis proceeds to vulnerable plaques. ATLO immune cell phenotypes, formation of conduits, HEVs, and aberrant lymph vessels indicate that hyperlipidemia-driven atherosclerosis involves significant innate and adaptive autoimmune responses. Mouse and human ATLOs may provide new experimental systems to isolate atherogenic autoantibodies and atherogenic T and B cell effectors and their antigen-specific counterparts carrying T and B cell receptors with specificity for epitopes of arterial wall-derived autoantigen(s). Studies of adventitial tissues could also facilitate identification of atherosclerosis-related autoantigens. Moreover, ATLOs may provide a new experimental model to identify the mechanisms how overt autoimmune atherosclerosis disease emerges from clinically silent autoimmune reactivity. This will require a considerably improved understanding of the functional impacts of adaptive immune cell subsets that act on the arterial wall. Delineation of the mechanisms underlying the disturbance of the equilibrium between these subsets may provide clues for translational research into human atherosclerosis and open avenues for immune-based therapeutics.

## Conflict of Interest Statement

The authors declare that the research was conducted in the absence of any commercial or financial relationships that could be construed as a potential conflict of interest.
